# An in vivo analysis of safe laparoscopic grasping thresholds for colorectal surgery

**DOI:** 10.1007/s00464-018-6172-6

**Published:** 2018-03-30

**Authors:** Jenifer Barrie, Louise Russell, Adrian J. Hood, David G. Jayne, Anne Neville, Peter R. Culmer

**Affiliations:** 1 School of Mechanical Engineering, The University of Leeds, Leeds, LS2 9JT UK; 2 Division of Clinical Sciences, Leeds Institute of Molecular Medicine, The University of Leeds, Leeds, UK

**Keywords:** Laparoscopy, Grasping, Colon

## Abstract

**Background:**

Analysis of safe laparoscopic grasping thresholds for the colon has not been performed. This study aimed to analyse tissue damage thresholds when the colon is grasped laparoscopically, correlating histological changes to mechanical compressive forces.

**Methods:**

An instrumented laparoscopic grasper was used to measure the forces applied to porcine colon, with data captured and plotted as a force–time (f–t) curve. Haematoxylin and eosin histochemistry of tissue subjected to 10, 20, 40, 50 and 70 N for 5, 30 and 60 s was performed, and the area of colonic circular and longitudinal muscle was compared in grasped and un-grasped regions. The area under the f–t curve was calculated as a measure of the accumulated force applied, known as the force–time product (FTP).

**Results:**

FTP ranged from 55.7 to 3793 N.s. Significant differences were observed between the muscle area of the grasped and un-grasped regions in both longitudinal and circular muscle at 50 N and above for all grasping times. For the longitudinal muscle, significant differences were observed between grasped and un-grasped areas at 20 N force for 30 s (mean difference = 59 mm^2^, 95% CI 41–77 mm^2^, *P* = 0.04), 20 N force for 60 s (mean difference = 31 mm^2^, 95% CI 21.5–40.5 mm^2^, *P* = 0.006) and 40 N force for 30 s (mean difference 37 mm^2^, 95% CI 27–47 mm^2^, *P* = 0.006). Changes in histology correlated with mechanical forces applied to the longitudinal muscle at a FTP over 300 N s.

**Conclusions:**

This study characterizes the grasping forces that result in histological changes to the colon and correlates these with a mechanical measurement of the applied force. The findings will contribute to the development of smart laparoscopic graspers with active constraints to prevent excessive grasping and tissue injury.

Little is known about the mechanics of the tool-tissue interaction in laparoscopic surgery and how it contributes to iatrogenic injury. Excessive grasping and retraction forces, long duration of grasps and the slip of the tissue in the grasper jaws may all contribute to tissue injury. In a systematic review of randomized controlled trials, Sammour et al. [1] found a higher rate of bowel injury and total intra-operative complications in laparoscopic colorectal operations compared to open resections. The risk of laparoscopic-induced bowel injury is reported to be as low as 0·13% [2], but up to 17.6% in more complex procedures [3]. In laparoscopic colorectal cancer operations, iatrogenic bowel injury is reported in 2% of colonic and 1% of rectal resections [2]. Although the majority of the grasper injuries are probably of minor clinical significance, the occurrence of a bowel perforation is a disastrous, yet largely avoidable, event. The mortality rate associated with laparoscopic-induced bowel injury is 3.6% [2] and increases with the complexity of the surgical procedure. Intra-operative tissue damage may lengthen operative time, result in a conversion to open surgery and increase patient morbidity [4]. The relationship between grasping force and inflammatory response, development of a paralytic ileus, and adhesion formation is not understood.

The aim of this study was to analyse the compressive forces involved in colonic grasping using a bespoke, instrumented surgical grasper and to correlate these forces with measures of tissue damage using histological analysis.

## Materials and methods

### Instrumented grasper system

**Fig. 1:**
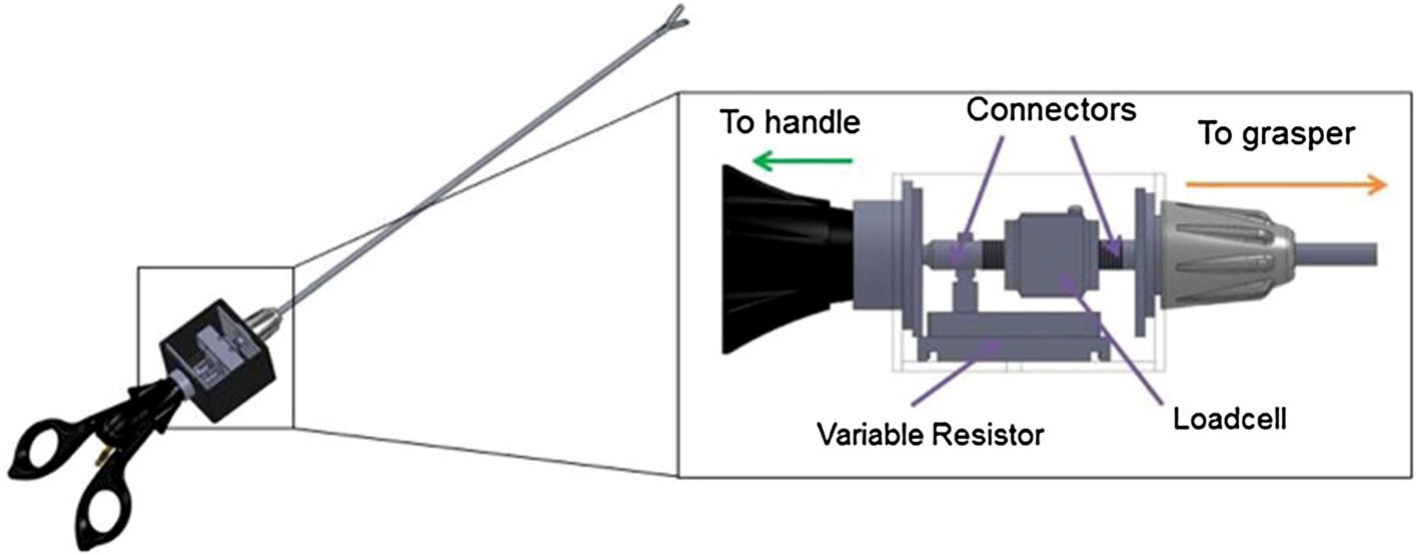
Diagram of instrumented grasper with instrumented module containing load cell and potentiometer

An instrumented grasper was developed by adapting a commercially available, reusable Johan grasper (Surgical Innovations Ltd UK) to integrate a bespoke sensor module at the instrument handle. The configuration positioned the electronic sensing elements outside the abdominal cavity, thus removing the risk of sensor contamination and ensuring that the tool–tissue interface is identical to that in a conventional grasper system (Fig. 1). The sensor module comprised a force sensor and a potentiometer position sensor connected to the instrument shaft that actuates the grasper jaws. A custom computer measurement system (LabVIEW, National Instruments Inc.) was used to measure and record data at 100 Hz. This enabled real-time measurement of the surgeon’s interaction with the instrument, linking instrument movement to forces generated at the grasper tip.

### Experimental protocol

The instrumented grasper was tested in a previously described in vivo porcine model [5]. All experiments were performed under Home Office licence (number PPL 40/3662) in a 40-kg Large White pig. The pig was acquired from the University of Leeds Animal Farm and allowed to acclimatize for 2 days prior to the experiment. General anaesthesia was induced using was induced using Propofol 10 mg/ml intravenously (4 mg/kg body weight or to effect). The pig was secured in the supine position on an operating table and access to the abdominal cavity achieved through a midline laparotomy. Manipulations were undertaken during a pre-specified surgical task on five different abdominal organs by a surgical research fellow who had completed a UK core surgical training programme.

### Force application

The range of forces applied to the bowel was based on results of in vivo bowel grasping experiments detailed in previous work by our group [5]. In this, the colon was grasped with the instrumented grasper and held without slip for 30 s. Four of these tasks were performed and the maximum force reached (F [max]) and the root mean squared force over the hold time, F [rms] were measured. Grasps were performed for 5, 30 and 60 s, consistent with both in vivo and ex vivo tissue experiments performed throughout the study and based on time-scales documented in the literature [6].

Grasps were performed on the anti-mesenteric border of the colon using the entire surface area of the instrumented grasper (surface area of one grasper jaw is 3.27E−5 M^2^). The pre-specified experimental parameters were five different forces (10, 20, 40, 50 and 70 N) applied for three time durations (5, 30 and 60 s). India ink staining was used to identify the grasped area of the tissue by applying it to the grasper jaws prior to grasping the tissue. A suture was placed between each grasped section in order to identify each sample correctly. On completion of the experiment, the pig was sacrificed by Schedule One killing. Each grasped segment was removed as a cylindrical piece with the grasped area identified using India ink.

### Histological analysis

A novel tissue damage assessment method was devised for this study after testing and optimization of two methods of measuring tissue damage, detailed in previous work by this group [7]. Haematoxylin and eosin staining was performed to analyse the tissue’s microscopic architecture and show evidence of physical tissue damage. The aim of these experiments was to examine the change in architecture of the colon as it is grasped in vivo. Histological analysis had to reflect this by blocking and cutting the sample in the configuration as they would be in vivo, a cylinder with the India ink on the outside representing the grasp. Samples were embedded in wax as a narrow cylinder as opposed to a flat single layer of colon Staining was performed following tissue blocking in wax, de-waxing and rehydration as per the protocol discussed in the author’s previous work [7]. Tissue was analysed using a Nikon E1000. Ten slides were generated for each experimental condition. Histopathological training was provided in both processing of slides and measurement of histological layers on the Nikon microscope by experienced technicians at Leeds Institute of Molecular Medicine. An experienced pathologist (Dr Nicholas West) was consulted in the process of evolving the methodology. Reviewer 1 (JB) devised the histological measurement methodology along with other authors (PC, DGJ and AN). Reviewer two was an experienced technician and was given a period of training in this particular measurement method.

**Fig. 2:**
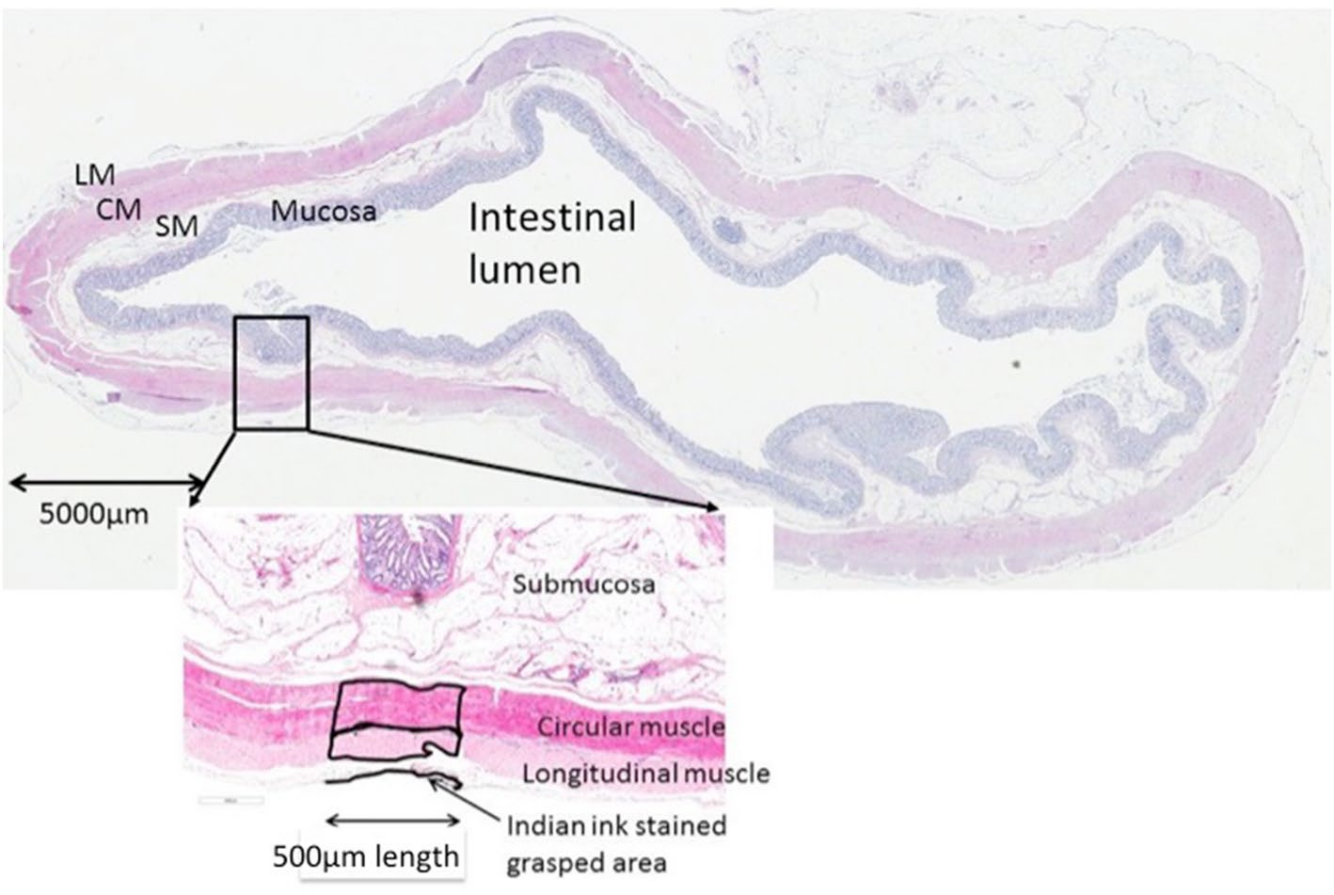
Measurement area as performed per protocol

Area measurements were taken over the most prominent area of India ink staining and the area of the longitudinal and circular muscle recorded within a 500 µm length (Fig. 2). Un-grasped (control) measurements were taken from a remote un-grasped area, with no evidence of India ink staining. This was performed manually using the measurement software, NIS Elements v2.2. No digital software could be identified to perform these measurements. Un-grasped measurements were combined as pooled data to compensate for biological variability in normal colonic muscle thickness, with the average value used for statistical comparison. Microscope measures were taken in micrometres but converted to millimetres for reporting to simplify the results. The area of the grasped circular and longitudinal muscle in each experimental condition was compared to the un-grasped measurement using a Student’s paired *t* test. Inter-rater and intra-rater variability was assessed by two independent assessors using the overall concordance correlation coefficient (OCCC) [8] on a subset of slides taken at a single variable (70 N 60 s) and representing 15% of the total number of measurements. Two observers (rater 1 and rater 2) blindly measured these histology slides. Rater 1 then re-measured the same slides again for comparison. Rater 1 therefore took two sets of measures, set 1a and 1b. The concordance correlation ranges between − 1 and 1, with a value of 1 corresponding to perfect agreement, a value of − 1 corresponding to perfect negative agreement, and a value of 0, corresponding to no agreement.

### Mechanical analysis

**Fig. 3:**
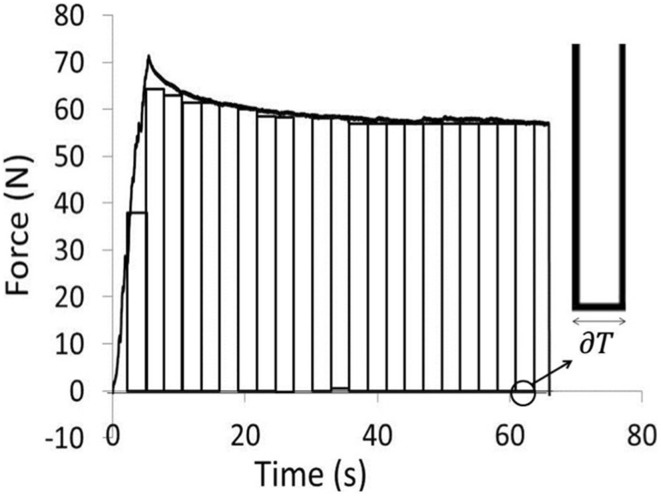
Schematic diagram showing method of calculating the area under the curve and therefore the force–time product (FTP)

Mechanical analysis was performed using a force–time representation of the data (Fig. 3). A relaxation profile was calculated by integrating the area under the force–time curve using Simpson’s rule [9]. There is currently no quantitative measure of tissue damage derived from mechanical data and this is an empirical measure of the accumulated force applied to the tissue (measured in N.s).

The following equation was used:

$${\text{Force}}{-}{\text{time product}}\left( {{\text{FTP}}} \right)={\text{sum}}\left( {\text{F}} \right) \times {\text{dT}},$$

where dT is the time interval between data samples, and sum (F) is the sum of all the forces reached over the manipulation time.

## Results

### Force application

Timing of the grasp would only commence once this force was reached; therefore, all F [max] results are above the pre-stipulated force. Timing of the pre-stipulated grasp commenced, when the pre-stipulated force was reached. The accuracy of these parameters was operator dependent. For purposes of this result section, each parameter will be described as it was pre-stipulated but there was variability in this as will be described here. The overall mean overshoot was 9.2 N (SD 9.8 N). Mean overshoot was 3.1 N for 60-s grasps, 6.7 N for 30-s grasps and 3 N at 5-s grasps. The higher overshoot at 30 s is reflected by the result for the 20 N grasp; the maximum force reached when grasping for 60 s was 22.1 N and for 5 s was 21.4 N but F [max] for the 30 s grasp reached more than double the stipulated grasping force at 46.2 N. These are shown in Table 1.

**Table 1:** F [max] for each grasp compared to the pre-stipulated force for the grasp

Pre-stipulated force (N)	F [max] reached for 60 s grasp (N)	F [max] reached for 30 s grasp (N)	F [max] reached for 5 s grasp (N)	Maximum overshoot (N)
70	71	72	72	2
50	52	51	57	7
40	42	42	43	3
20	22	46	21	26
10	18	12	12	8

### Histological analysis

**Fig. 4:**
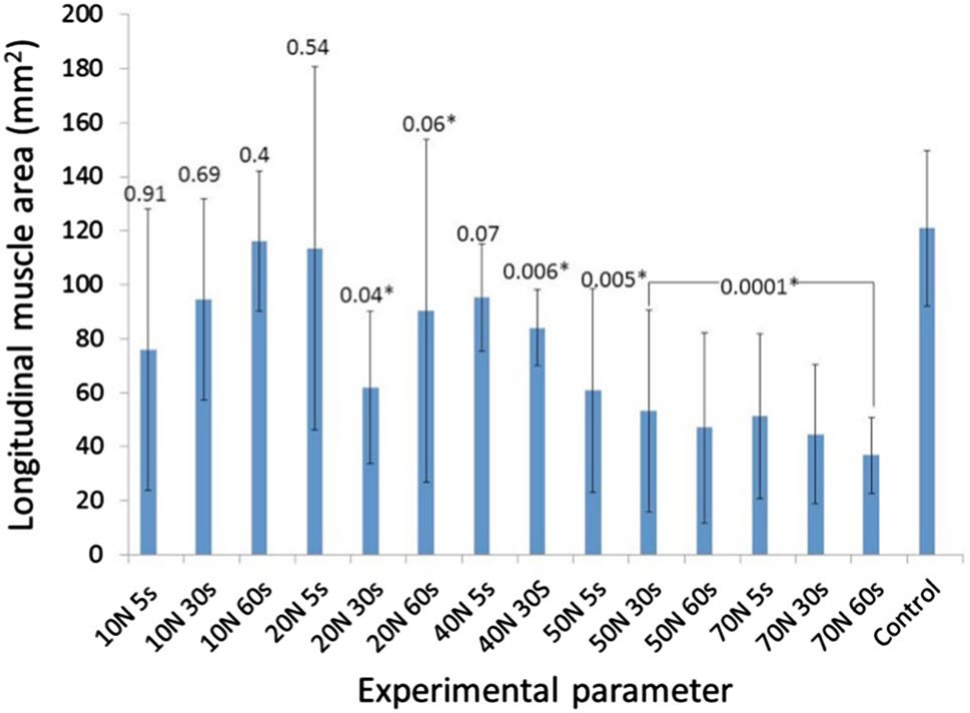
Graph showing grasped versus un-grasped measures for the longitudinal muscle with *P* values shown above each parameter

**Fig. 5:**
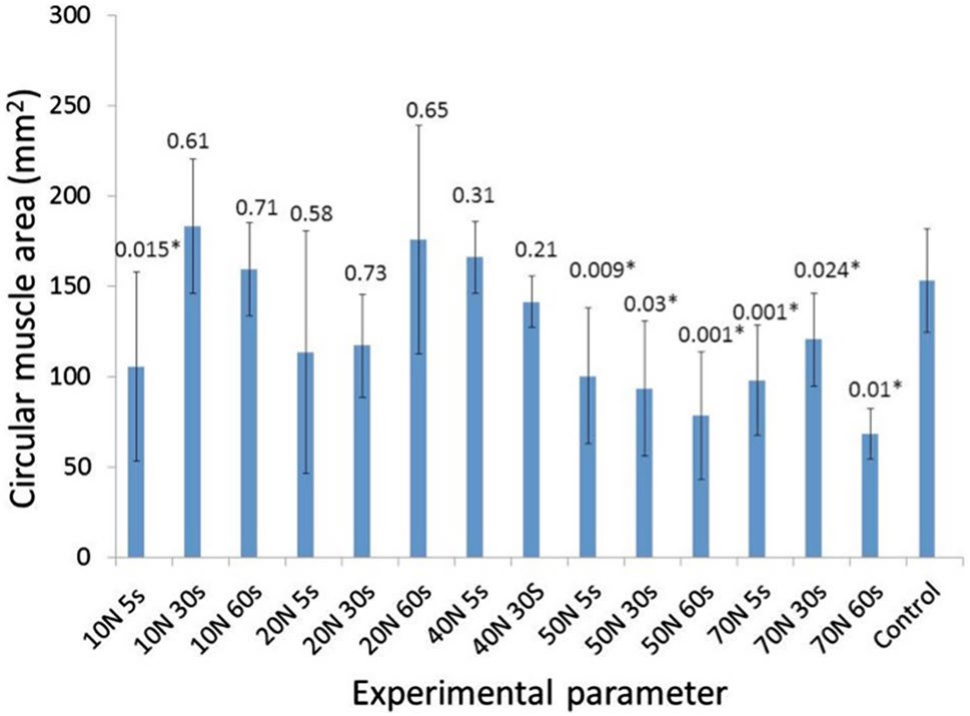
Graph showing grasped versus un-grasped measures for the circular muscle with *P* values shown above each parameter

The combined un-grasped measure was 153 mm^2^ (± 28.7 mm^2^) for the circular muscle and 121 mm^2^ (± 57 mm^2^) for the longitudinal muscle. Statistically significant differences were observed between the muscle area of the grasped and un-grasped regions in both longitudinal and circular muscle at 50 N and above for all three grasping durations. For the longitudinal muscle, significant differences were observed between grasped and un-grasped areas at 20 N force for 30 s (mean difference = 59 mm^2^, 95% CI 41–77 mm^2^, *P* = 0.04), 20 N force for 60 s (mean difference = 31 mm^2^, 95% CI 21.5–40.5 mm^2^, *P* = 0.006) and 40 N force for 30 s (mean difference 37 mm^2^, 95% CI 27–47 mm^2^, *P* = 0.006). A significant difference was found between the grasped and un-grasped circular muscle at 10 N 5 s (mean difference 47 mm^2^, 95% CI 36.1–57.9 mm^2^, *P* = 0.015). Measurements from the grasped and un-grasped areas of longitudinal muscle and grasped and un-grasped circular muscle under the various experimental conditions are shown in Figs. 4 and 5, respectively.

The results of agreement between 1a, 1b and 2 in each group are presented in Table 2. The grasped tissue groups had higher OCCC values than the un-grasped groups. All the OCCCs were significant (from zero or no agreement), except the un-grasped circular group. The correlation in the grasped section was higher than that of the un-grasped section for both circular (0.796 vs. 0.287) and longitudinal muscle (0.778 vs. 0.487).

**Table 2:** Concordance correlation coefficient for the overall concordance measurements between observations 1a, 1b and 2

	Overall CCC (OCCC)	95% confidence interval
Grasped longitudinal muscle	0.778	(0.199, 0.908)
Un-grasped longitudinal muscle	0.487	(0.024, 0.757)
Grasped circular muscle	0.796	(0.377, 0.915)
Un-grasped circular muscle	0.287	(− 0.046, 0.556)

### Linking mechanical and histological analysis

**Fig. 6:**
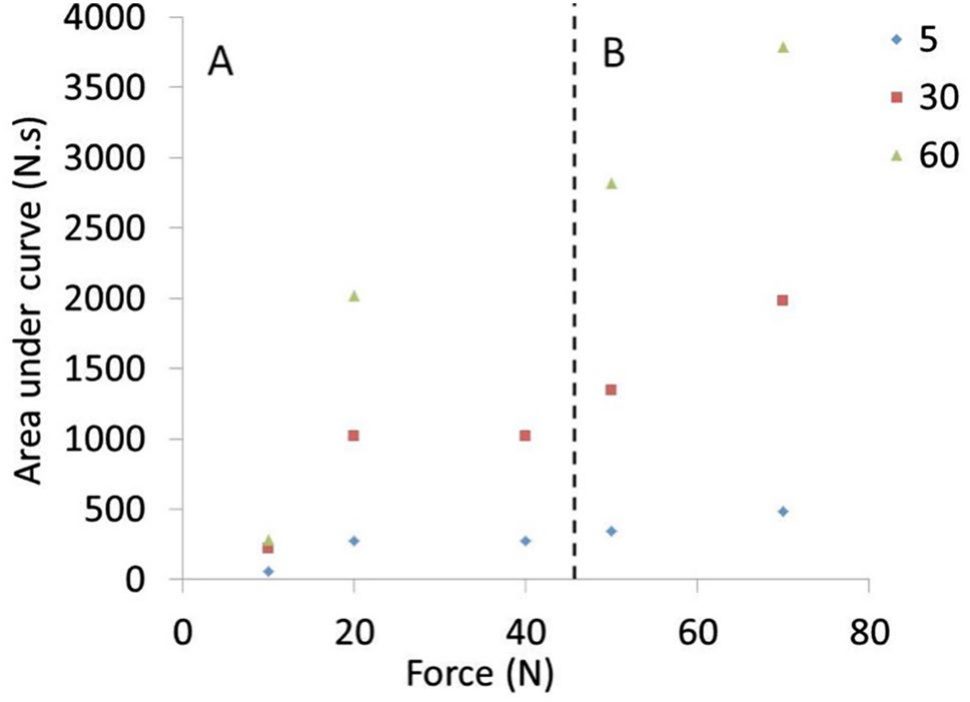
FTP plotted for all parameters. A dashed line separates region **A** and region **B**. Region **B** denotes the parameters where a statistically significant difference was found between both the circular and the longitudinal muscle measures and their corresponding un-grasped regions

Statistically significant differences between the muscle area of the grasped and un-grasped regions in both longitudinal and circular muscle were found at 50 N and above for all three grasping times. Figure 6 shows the FTP plotted for each parameter divided into two regions, region A and region B, separated by a dashed line. Region B denotes the parameters where a statistically significant difference was found between both the circular and the longitudinal muscle measures and their corresponding un-grasped regions. The largest FTP in region A was 1017 N.s (20 N 30 s). The largest FTP in region B was 343 N.s (50 N 5 s). For the longitudinal muscle, statistically significant differences between grasped and un-grasped longitudinal muscle areas were found above 20 N 30 s. All significant histological results corresponded with a FTP value of over 300 N.s. The 40 N 5 s result, which was non-significant, was 271 N.s in comparison to 50 N 5 s which was 343 N.s. The one exception to this was the result at 10 N 5 s (*P* = 0.015) with a FTP of 56 N.s.

## Discussion

This study characterizes the grasping forces that result in histological change to pig colon tissue in vivo and, for the first time, correlates this with a mechanical measurement. The experiments presented contribute to understanding and quantifying the tool-tissue interaction in minimally invasive surgery, and provide an experimental methodology for future research. The limitations of this study are the use of a single pig and constraining experimental variables to a single laparoscopic grasper type operated by a single surgeon. The single pig reflects the scope of our preliminary work and the need to demonstrate a methodology to assess the tool–tissue interaction. Additionally, ethical considerations dictate that an animal model is used prior to experimentation in humans. Time constraints in conducting the in vivo experiments limited the range of experimental conditions. In vivo testing was performed in a 40-kg Large White pig because the intestinal size at this weight resembles the adult human. The Johan grasper was selected because it is commonly used in a wide variety of laparoscopic procedures. The eventual aim is to broaden the scope of our research to include other instruments and mechanisms. For example, the “parallel occlusion mechanism” aims to generate even pressure distribution on the tissues being grasped and less trauma to the tissues [10]. Further testing should include these graspers.

In the concordance measurements for histological change, the grasped sections generally had higher OCCC than the un-grasped sections. The agreement was generally non-significant or borderline significant in the un-grasped groups. This can be explained by the fact that the methodology stipulated that measurements were taken from an area remote to the Indian ink. Natural biological variability in muscle thickness at various points has resulted in a low correlation coefficient in the control measurements. In optimizing the methodology for further work, a fixed control point on each slide should be identified to be measured by both raters. It is imperative to have consistent and repeatable results in studies of tissue damage and other studies of tissue damage have not included concordance measures [11, 12]. To achieve repeatable, measurable results, alternative measurement software methods would be optimal but we were unable to identify specialized digital software for this particular methodology.

Force application in this study was based on previous published results from this group [5]. In these data, the range of F [max] was between 43 and 76 N. Mean F [rms] was 25 N [5]. Although the mean F [rms] was 25, 10 N was the lowest force applied. Other studies have demonstrated lower manipulation forces resulting in tissue damage [6]. A mean perforation force of 13.5 N for the large bowel was identified by Heijnsdijk et al. [7] in a study investigating safety margins for laparoscopic forces. The highest F [max] of 76 N was slightly higher than the largest force applied in these experiments of 70 N. Other groups have measured grasping force intra-operatively [13, 14]. Hanna et al. [14] developed a system to measure the gripping, dissecting, pulling and pushing forces as well as the force vector at a port site and determining the position of instrument’s jaws using sensors mounted on the forceps handle. This study did not include results on tissue manipulation forces. Yoshida et al. [13] recorded the force pattern of a single maneuver and measured instrument tip forces. Correlating measured force with histological change to the colon has not been reported in the literature.

An alternative method of determining damage may have been to devise a tissue damage grading system, akin to that devised by Marucci et al. [15] for the gallbladder wall, by Li et al. [16] in porcine liver, or Miyasaka et al. [17] in porcine small bowel. A method of grading macroscopic tissue damage was devised by Vonck et al. [18] in their experimental study of a novel vacuum grasping method. Miyasaka et al. [17] also developed a tissue damage grading system to be used after histological processing on the small bowel. Vonck’s method of grading macroscopic tissue injury is novel and specific for the grasper used—no macroscopic tissue damage was observed. This may be because the bowel was left in vivo for four hours and therefore any indentation left on the serosa of the bowel recovered. Intuitively, there will be grasping conditions that do result in tears of the serosa or perforation of the bowel. The most comprehensive study analysing the effects of mechanical stress on tissue was in the thesis work of De [19], which provides a novel approach to damage assessment and was the first time that quantitative damage assessment and measures other than purely qualitative structural analysis were performed. This group used a motorized endoscopic grasper fitted with an atraumatic Babcock grasper to apply compression stresses to the small bowel, ureter and liver. The morphology and architecture of the tissue were assessed qualitatively, and immunohistochemical evidence of neutrophil infiltration and apoptosis was used as markers of inflammation and tissue damage. Immunohistochemical analysis of inflammatory cell infiltrate takes time to develop post-injury. Macrophages accumulate at the site of injury after a few hours, followed by neutrophils between 4 and 6 h. It can take up to 24 h for appreciable cellular accumulation. The time constraints of our study did not allow us to study these inflammatory aspects of tissue injury. Instead, we have relied on histological analysis to measure tissue damage because it gave a reliable method for assessing change in muscle area. This minimizes errors in comparison to using single-point tissue width measures. While the use of histopathology will always be susceptible to biological variation and processing artefacts, it also provides a standardized technique with controlled protocols which could be readily adopted by other researchers.

Further work has been carried out in our institution in defining mechanical damage thresholds ex vivo. A metric which considers the rate at which stress is increasing in the tissue and normalizes this with respect to the loading rate (the speed at which the grasper jaws are closing) has been devised and tested in ex vivo conditions. This takes into account load rate and load history and will be important in developing a more sophisticated metric for tissue damage thresholds [20].

Handling the bowel is obviously unavoidable during laparoscopic surgery, but this study shows that a combination of increased grasping force and longer grasp times increase the risk of irreversible tissue change. Surgeons should avoid prolonged grasps and grasping with high pressure at the handle, but maintain enough force to prevent slip from the grasper jaws. The current study has successfully identified specific loading conditions that result in tissue injury and is the first to establish an important link between the mechanical analyses of tissue manipulation with change to the architecture of the tissue. The methodology and data presented will contribute to the development of smart laparoscopic graspers with active constraints to prevent excessive grasping and tissue injury with the goal of improving surgical safety and morbidity.
